# Correlations between retinal thickness, brain and blood biomarkers in preclinical Alzheimer's disease: pilot

**DOI:** 10.1002/alz70856_102434

**Published:** 2025-12-25

**Authors:** Isaiah‐Lorenzo De Guia, Shaun Eslick, Swathi Kanduri, Purna Poudel, Eugene Hone, Ralph N Martins

**Affiliations:** ^1^ Macquarie University, Sydney, NSW, Australia; ^2^ Macquarie University, Macquarie Park, NSW, Australia; ^3^ Australian Alzheimer's Research Foundation, Perth, Western Australia, Australia; ^4^ Edith Cowan University, Perth, Western Australia, Australia; ^5^ Lions Alzheimer's Foundation, Perth, Western Australia, Australia; ^6^ Alzheimer's Research Australia, Perth, Western Australia, Australia

## Abstract

**Background:**

Retinal deposits of amyloid beta (Aβ) have been identified in the eye and has shown to have a greater impact on retinal thickness than age‐related effects. Therefore, retinal thickness could serve as a diagnostic marker, warranting further investigation as demand for accessible Alzheimer's disease (AD) screening grows. This study aims to evaluate correlations between blood‐based AD biomarkers and retinal thickness across different eye regions in amyloid‐negative and amyloid‐positive individuals.

**Method:**

Optical coherence tomography (OCT) and AD plasma biomarkers NFL, Aβ42, Aβ40, GFAP were analysed in cognitively normal individuals (MMSE >24) aged 58‐84 years, who previously undertook Positron Emission Tomography (PET) amyloid imaging. A centiloid (CL) cut‐off score of 20 was used to subgroup recruited participants into amyloid‐positive (*n* = 5) and amyloid‐negative (*n* = 5). Data was analysed in reference to eye (LE/RE), image location (optic disc, macula) and ETDRS (early treatment diabetic retinal study) regions. R software was used to calculate Spearman correlation coefficients, with a significance level of *p* < 0.05.

**Result:**

Comparing correlation coefficients across both groups demonstrated the amyloid‐negative group having strong negative correlations between CL scores and total retinal thickness in only the peripapillary region (*r* = ‐0.71 to ‐0.97, *p* =  <0.05), where the amyloid‐positive group had negative correlations in both macular and peripapillary regions (*r* = ‐0.71 to –1, *p* =  <0.05). The amyloid‐negative group exhibited strong positive correlations with macular (*r* = 0.8 to 0.94, *p* =  <0.05) and outer disc peripapillary (*r* = 0.7, *p* =  <0.05) thickness to the Aβ42/Aβ40 ratio. The amyloid‐positive groups retinal thicknesses across various regions had stronger negative associations of GFAP and NFL (*r* = ‐0.7 to ‐0.9, *p* =  <0.05) than the amyloid‐negative group (*r* = ‐0.63 to ‐0.8, *p* =  <0.05).

**Conclusion:**

These findings suggest that total retinal thickness in the macular and peripapillary regions via OCT may correlate with AD biomarkers. Increased retinal thickness were associated with increased Aβ42/AΒ40 ratio and decreased retinal thickness associated with higher brain amyloid CL scores, and blood‐based GFAP and NFL biomarkers. Further study in larger cohorts is required to validate results.

